# Crystal structure, Hirshfeld surface analysis and geometry optimization of 2-hy­droxy­imino-*N*-[1-(pyrazin-2-yl)ethyl­idene]propano­hydrazide

**DOI:** 10.1107/S2056989022007927

**Published:** 2022-08-12

**Authors:** Maksym O. Plutenko, Svitlana V. Shishkina, Oleg V. Shishkin, Vadim A. Potaskalov, Valentina A. Kalibabchuk

**Affiliations:** a Department of Chemistry, National Taras Shevchenko University, Volodymyrska Street 64, 01601 Kyiv, Ukraine; b "Institute for Single Crystals" NAS of Ukraine, 60 Nauky ave., Kharkiv, 61001, Ukraine; c V. N. Karazin Kharkiv National University, 4 Svobody sq., Kharkiv 61022, Ukraine; d Department of General and Inorganic Chemistry, National Technical University of Ukraine, ‘Kyiv Polytechnic Institute’, 37 Prospect Peremogy, 03056 Kiev, Ukraine; e Department of Analytical, Physical and Colloid Chemistry, O. O. Bohomolets National Medical University, Shevchenko Blvd. 13, 01601 Kiev, Ukraine; Katholieke Universiteit Leuven, Belgium

**Keywords:** crystal structure, hydrazide, hydrazone, oxime, Schiff base, polynucleative ligand

## Abstract

In the mol­ecule of the title compound, C_9_H_11_N_5_O_2_, the oxime and hydrazide groups are situated in a *cis*-position in relation to the C—C bond linking the two functional groups. The CH_3_C(=NOH)C(O)NH fragment deviates from planarity because of a twist between the oxime and amide groups. In the crystal, mol­ecules are linked by O—H⋯O hydrogen bonds, forming zigzag chains in the [013] and [0  3] directions.

## Abstract 2

The title compound, 2-hy­droxy­imino-*N*-[1-(2-pyrazin­yl)ethyl­idene]propane­hydrazide, is a ligand able to form polynuclear metal complexes. The mol­ecule is not planar due to a twist between the oxime and amide groups. In the crystal, mol­ecules are linked by O—H⋯O hydrogen bonds into supra­molecular chains.

## 1. Chemical context

The combination in one mol­ecule of two donor sets of a different nature, such as oxime and hydrazide, might be the key to creating new asymmetric polynucleative ligands suitable for the formation of polynuclear complexes. In recent decades, a number of ligands based on 2-hy­droxy­imino­propane­hydrazide have been obtained. It was shown that such a type of ligand reveals a strong tendency for the formation of polynuclear complexes (Anwar *et al.*, 2011 ▸, 2012 ▸; Fritsky *et al.*, 2006 ▸; Jin *et al.*, 2022 ▸). 
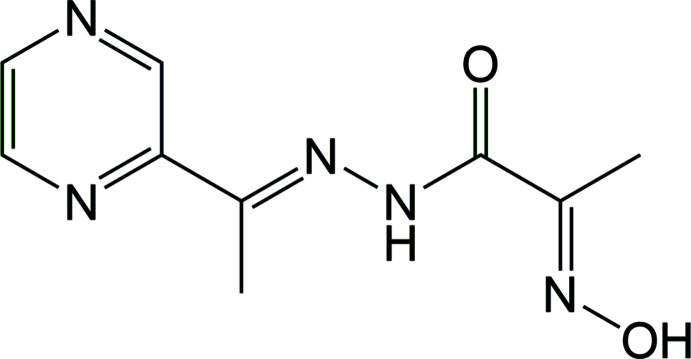


The title compound, 2-hy­droxy­imino-*N*-[1-(pyrazin-2-yl)ethyl­idene]propano­hydrazide (**1**), was first described in the work of Feng and co-workers (Feng *et al.*, 2018 ▸). It acts as a ligand in three new polynuclear heterometal porous coordination polymers, which have displayed high CO_2_ adsorption uptake and high adsorption selectivity of CO_2_ over N_2_ and CH_4_. The present work is devoted to the synthesis, crystal structure, spectroscopic characterization, Hirshfeld surface analysis and quantum mechanical geometry optimization of **1**.

## 2. Structural commentary

**Figure 1:**
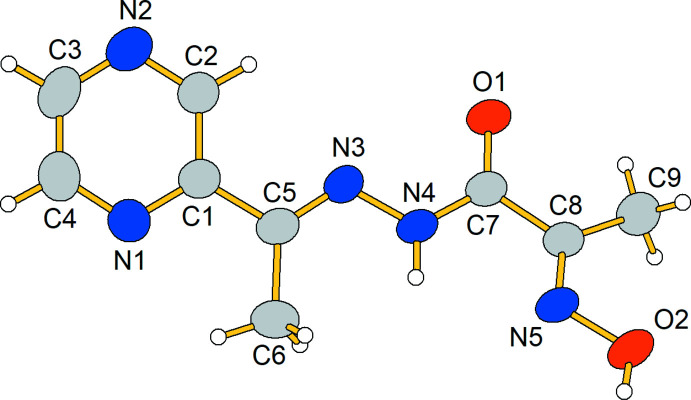
The mol­ecular structure of the title compound **1** with displacement ellipsoids shown at the 50% probability level.

The title compound, **1**, crystallizes in space group *Pca*2_1_ (Fig. 1 ▸). The N—O and C—N bond lengths of the oxime group are 1.382 (3) and 1.278 (4) Å, respectively, which is typical for neutral moieties of this type (Fritsky *et al.*, 1998 ▸, 2004 ▸). The N—N, N—C and C—O bond lengths of the hydrazide group [1.370 (3), 1.332 (4) and 1.229 (4) Å, respectively] are typical for 2-(hy­droxy­imino)­propane­hydrazide derivatives (Hegde *et al.*, 2017 ▸; Malinkin *et al.*, 2012 ▸; Moroz *et al.*, 2009*a* ▸,*b* ▸; Plutenko *et al.*, 2011 ▸). The oxime and the hydrazide groups are situated in a *cis*-position about the C7—C8 bond, which is also typical for 2-(hy­droxy­imino)­propane­hydrazide derivatives. Such a conformation is stabilized additionally by an H4⋯N5 attractive inter­action (2.33 Å). Despite the distance being shorter than the sum of the van der Waals radii (2.67 Å; Zefirov, 1997 ▸) the inter­action cannot be classified as an intra­molecular hydrogen bond because of the acute N4—H⋯N5 angle (101°).

The CH_3_C(=NOH)C(O)NH fragment deviates from planarity (r.m.s. deviation of 0.362 Å) because of a twist between the oxime and the amide groups about the C7—C8 bond. The maximum deviations are 0.8763 (9) and 0.3355 (18) Å, respectively, for hydrogen (H9*C*) and non-hydrogen (O1) atoms. The O1—C7—C8—N5 torsion angle is 165.1 (3)°, significantly less than the average value in 2-(hy­droxy­imino)­propane­hydrazide derivatives published previ­ously [172.1 (4)°]. Thus, such a twist distortion of the mol­ecule seems to be a result of the crystal packing.

## 3. Supra­molecular features

**Figure 2:**
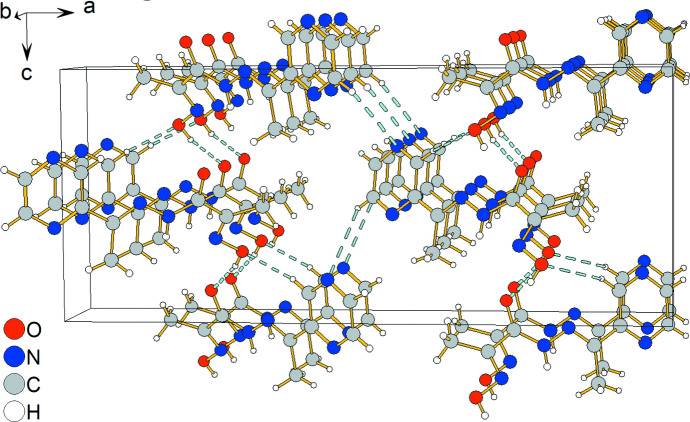
Crystal packing of the title compound **1**. Hydrogen bonds are indicated by dashed lines.

In the crystal, mol­ecules are linked by O2—H2⋯O1^i^ and C2—H2*A*⋯O2^ii^ inter­molecular hydrogen bonds [symmetry codes: (i) −*x* + 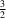 , *y* + 1, *z* + 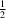 ; (ii) −*x* + 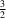 , *y* − 1, *z* − 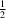 ], forming zigzag chains in the [013] and [0 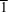 3] crystallographic directions (Fig. 2 ▸). These chains alternate in the [100] direction and are linked by C4—H4*A*⋯N2^iii^ inter­molecular hydrogen bonds [symmetry code: (iii) −*x* + 1, −*y* − 1, *z* + 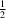 ]. Details of the hydrogen-bond geometry are given in Table 1 ▸.

## 4. Hirshfeld surface analysis

**Figure 3:**
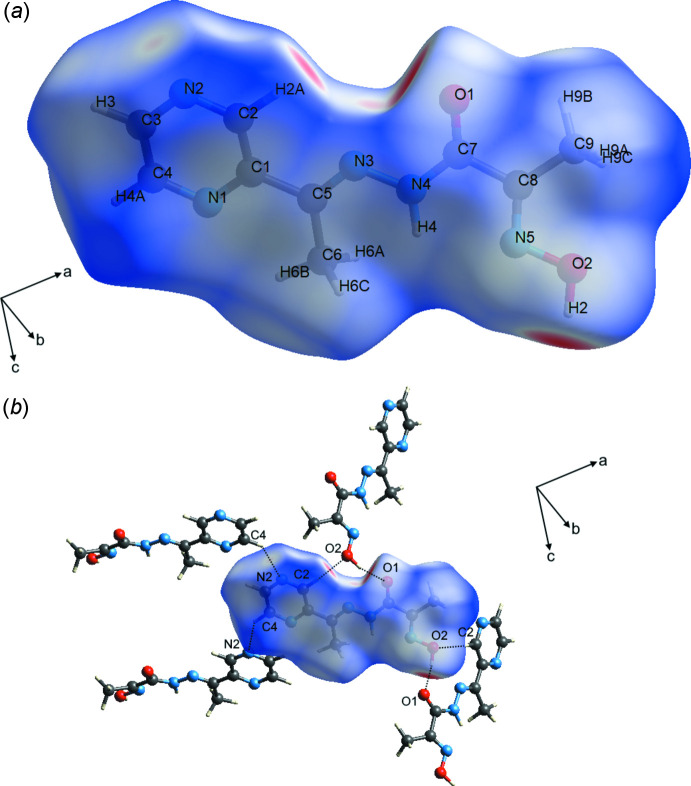
The Hirshfeld surface of the title mol­ecule **1** mapped over *d*_norm_, showing the close contacts.

The Hirshfeld surface analysis (Spackman & Jayatilaka, 2009 ▸) and the associated two-dimensional fingerprint plots (McKinnon *et al.*, 2007 ▸) were performed with *CrystalExplorer17* (Turner *et al.*, 2017 ▸). The Hirshfeld surfaces of the complex anions are colour-mapped with the normalized contact distance (*d*_norm_) from red (distances shorter than the sum of the van der Waals radii) through white to blue (distances longer than the sum of the van der Waals radii). The Hirshfeld surface of the title compound mapped over *d*_norm_, in the colour range −0.6441 to 1.3084 a.u. is shown in Fig. 3 ▸. According to the Hirshfeld surface, O2—H2⋯O1 and C4—H4*A*⋯N2 are the most noticeable inter­molecular inter­actions. In addition, a C2—H2*A*⋯O2 weak inter­molecular inter­action is observed.

**Figure 4:**
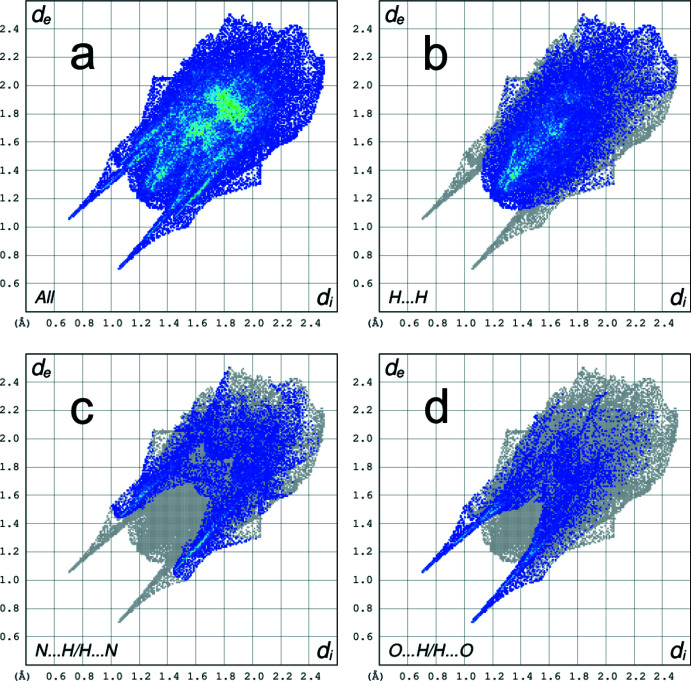
A view of the two-dimensional fingerprint plots for the title compound **1** showing (*a*) all inter­actions, and delineated into (*b*) H⋯H (41.9%), (*c*) N⋯H/H⋯N (20.5%) and (*d*) O⋯H/H⋯O (15.4%) contacts.

A fingerprint plot delineated into specific inter­atomic contacts contains information related to specific inter­molecular inter­actions. The blue colour refers to the frequency of occurrence of the (*d*_i_, *d*_e_) pair with the full fingerprint plot outlined in grey. Fig. 4 ▸ shows the two-dimensional fingerprint plots of the sum of the contacts contributing to the Hirshfeld surface represented in normal mode. The most significant contribution to the Hirshfeld surface is from H⋯H (41.9%) contacts. In addition, N⋯H/H⋯N (20.5%) and O⋯H/H⋯O (15.4%) are highly significant contributions to the total Hirshfeld surface. The O⋯H/H⋯O fingerprint plot (Fig. 4 ▸ *d*) reveals two sharp spikes along 1.9 Å < *d_i_* + *d_e_* < 2.4 Å, which are associated with the O2—H2⋯O1 hydrogen bond.

## 5. Geometry optimization

**Figure 5:**
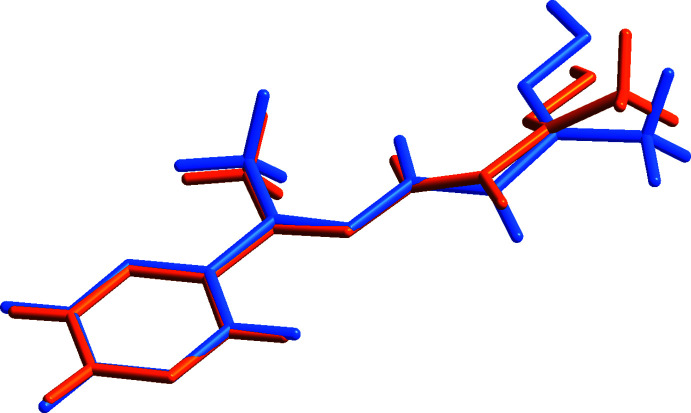
Overlay between the mol­ecule obtained from experimental (orange) and DFT optimization (blue).

The DFT quantum-chemical calculations were performed at the B3LYP/6-311 G(d,p) level (Becke, 1993 ▸) as implemented in *PSI4* software package (Parrish *et al.*, 2017 ▸). The GFN2-xTB (Bannwarth *et al.*, 2019 ▸) calculations were applied with *xtb 6.4* package (Grimme, 2019 ▸). The structure optimization of the title compound was performed starting from the X-ray geometry and the resulting geometric values were compared with experimental values (Table 2 ▸, Fig. 5 ▸). The r.m.s. deviations are 0.380 and 0.362 Å for DFT and GFN2-xTB, respectively.

The calculated geometric parameters are in good agreement with experimental values. It is important to note that the accuracy of the semi-empirical GFN2-xTB method is close to that of the DFT calculations, even though GFN2-xTB calculations are significantly computationally ‘cheaper’ (∼2·10^3^ times faster for the calculations described here).

The most significant difference between the calculated and X-ray geometries is the absence of a twist deformation between the oxime and the amide groups in the case of QM calculated geometries. This might be additional evidence that the twist distortion of the mol­ecule is due to effects of the crystal packing. The largest differences between the X-ray and calculated bond lengths are observed for the hydrazide moiety: N3—N4 is slightly longer (0.019 and 0.034 Å for DFT and GFN2-xTB, respectively) and C7—N4 is shorter (0.050 and 0.036 Å for DFT and GFN2-xTB, respectively) than calculated. Such calculation errors are probably typical for hydrazide derivatives at this level of theory (Anitha *et al.*, 2019 ▸; Malla *et al.*, 2022 ▸). The HOMO–LUMO gap calculated by DFT method is 0.159 a.u. and the frontier mol­ecular orbital energies, *E*_HOMO_ and *E*_LUMO_ are −0.23063 and −0.07178 a.u., respectively.

## 6. Database survey

A search in the Cambridge Structural Database (CSD version 5.43, update of March 2022; Groom *et al.*, 2016 ▸) resulted in seven hits for 2-(hy­droxy­imino)­propane­hydrazide derivatives: CUDBEJ, DUDHOA, OBUXIU, PUVPED, PUVPED01, WARCEZ and WARCID (Hegde *et al.*, 2017 ▸; Malinkin *et al.*, 2012 ▸; Moroz *et al.*, 2009*a* ▸,*b* ▸; Plutenko *et al.*, 2011 ▸). Most of them deviate slightly from planarity: r.m.s. deviations are in the range 0.247-0.390 Å with maximum deviations of non-hydrogen atoms from the best plane in the range 0.098–0.340 Å. At the same time PUVPED and PUVPED01 are not planar, mainly because of a twist of the di­carbonyl­hydrazine group [the C—N—N—C torsion angle is 96.54 (15)°].

157 hits relate to organometallic substances based on 2-(hy­droxy­imino)­propane­hydrazide derivatives. Most of them are polynuclear 3*d* and 4*f* metal complexes (discrete mol­ecules and MOFs). The maximum number of metal centres per mol­ecule for the discrete complexes of this type is 12 (Anwar *et al.*, 2011 ▸, 2012 ▸; Moroz *et al.*, 2012 ▸).

## 7. Synthesis and crystallization

The title compound was prepared according to a slightly modified procedure (Feng *et al.*, 2018 ▸). A solution of 2-(hy­droxy­imino)­propane­hydrazide (0.702 g, 5 mmol) in methanol (50 ml) was treated with 2-acetyl­pyrazine (0.732 g, 5 mmol) and the mixture was heated under reflux for 1.5 h. After that, the solvent was evaporated under vacuum and the product was recrystallized from methanol. Yield 1.141 g (86%). ^1^H NMR, 400.13 MHz, (DMSO-*d*_6_): 11.97 (*s*, 1H, OH), 10.21 (*s*, 1H, NH), 9.31 (*s*, 1H, pyrazine-3), 8.56 (*s*, 1H, pyrazine-5), 8.55 (*s*, 1H, pyrazine-6), 2.37 (*s*, 3H, hydrazonic CH_3_), 2.02 (*s*, 3H, CH_3_). IR (KBr, cm^−1^): 1658 (CO amid I), 1034 (NO oxime). Analysis calculated for C_9_H_11_N_5_O_2_: C 48.86, H 5.01, N 31.66%; found: C 48.49, H 5.22, N 31.42%.

## 8. Refinement

Crystal data, data collection and structure refinement details are summarized in Table 3 ▸. All the hydrogen atoms were positioned geometrically (N—H = 0.85, C—H = 0.93–0.96 Å) and refined using a riding model with *U*_iso_ = *nU*_eq_ of the carrier atom (*n* = 1.5 for methyl groups and *n* = 1.2 for other hydrogen atoms).

## Supplementary Material

Crystal structure: contains datablock(s) I. DOI: 10.1107/S2056989022007927/vm2270sup1.cif

Structure factors: contains datablock(s) I. DOI: 10.1107/S2056989022007927/vm2270Isup2.hkl

Supporting information file. DOI: 10.1107/S2056989022007927/vm2270Isup3.cdx

CCDC reference: 2195126

Additional supporting information:  crystallographic information; 3D view; checkCIF report

## Figures and Tables

**Table 1:** Hydrogen-bond geometry (Å, °)

*D*—H⋯*A*	*D*—H	H⋯*A*	*D*⋯*A*	*D*—H⋯*A*
O2—H2⋯O1^i^	0.82	1.94	2.741 (3)	167
C2—H2*A*⋯O2^ii^	0.93	2.35	3.243 (5)	161
C4—H4*A*⋯N2^iii^	0.93	2.67	3.451 (6)	142

Symmetry codes: (i) 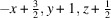 ; (ii) 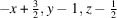 ; (iii) 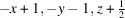 .

**Table 2:** Comparison of selected geometric data (A,°) from calculated and X-ray data

	X-ray	DFT	GFN2-xTB
Oxime moiety			
C8=N5	1.278 (4)	1.285	1.273
N5—O2	1.382 (3)	1.394	1.389
C8—N5—O2	111.4 (2)	112.1	116.0
Hydrazide moiety			
C7=O1	1.229 (4)	1.218	1.208
C7—N4	1.332 (4)	1.382	1.368
N3—N4	1.370 (3)	1.351	1.336
O1—C7—N4	124.1 (3)	124.6	124.7
Other			
C5=N3	1.278 (4)	1.292	1.279
O1—C7—C8—N5	165.1 (3)	179.9	179.0

**Table 3:** Experimental details

Crystal data	
Chemical formula	C_9_H_11_N_5_O_2_
*M*_r_	221.23
Crystal system, space group	Orthorhombic, *P* *c* *a*2_1_
Temperature (K)	293
*a*, *b*, *c* (Å)	24.367 (2), 4.3979 (5), 10.1424 (9)
*V* (Å^3^)	1086.89 (18)
*Z*	4
Radiation type	Mo *K*α
μ (mm^−1^)	0.10
Crystal size (mm)	0.8 × 0.4 × 0.1

Data collection	
Diffractometer	Xcallibur3
Absorption correction	Multi-scan (*CrysAlis PRO*; Rigaku OD, 2019 ▸)
*T*_min_, *T*_max_	0.646, 1.000
No. of measured, independent and observed [*I* > 2σ(*I*)] reflections	2381, 1540, 1254
*R*_int_	0.023
(sin θ/λ)_max_ (Å^−1^)	0.595

Refinement	
*R*[*F* ^2^ > 2σ(*F* ^2^)], *wR*(*F* ^2^), *S*	0.037, 0.088, 1.01
No. of reflections	1540
No. of parameters	148
No. of restraints	1
H-atom treatment	H-atom parameters constrained
Δρ_max_, Δρ_min_ (e Å^−3^)	0.11, −0.13
Absolute structure	Flack *x* determined using 351 quotients [(*I* ^+^)−(*I* ^−^)]/[(*I* ^+^)+(*I* ^−^)] (Parsons *et al.*, 2013 ▸)
Absolute structure parameter	−1.7 (10)

Computer programs: *CrysAlis PRO* (Rigaku OD, 2019 ▸), *SHELXT* (Sheldrick, 2015*a* ▸), *SHELXL2016/6* (Sheldrick, 2015*b* ▸), *DIAMOND* (Brandenburg, 2009 ▸) and *OLEX2* (Dolomanov *et al.*, 2009 ▸).

## supplementary crystallographic information

### Crystal data

**Table:**

C_9_H_11_N_5_O_2_	*D*_x_ = 1.352 Mg m^−^^3^
*M**_r_* = 221.23	Mo *K*α radiation, λ = 0.71073 Å
Orthorhombic, *P**c**a*2_1_	Cell parameters from 1825 reflections
*a* = 24.367 (2) Å	θ = 2.4–25.3°
*b* = 4.3979 (5) Å	µ = 0.10 mm^−^^1^
*c* = 10.1424 (9) Å	*T* = 293 K
*V* = 1086.89 (18) Å^3^	Plate, colourless
*Z* = 4	0.8 × 0.4 × 0.1 mm
*F*(000) = 464	

### Data collection

**Table:**

Xcallibur3 diffractometer	1254 reflections with *I* > 2σ(*I*)
area detector scans	*R*_int_ = 0.023
Absorption correction: multi-scan (CrysAlisPro; Rigaku OD, 2019)	θ_max_ = 25.0°, θ_min_ = 3.3°
*T*_min_ = 0.646, *T*_max_ = 1.000	*h* = −13→28
2381 measured reflections	*k* = −5→4
1540 independent reflections	*l* = −12→10

### Refinement

**Table:**

Refinement on *F*^2^	Hydrogen site location: mixed
Least-squares matrix: full	H-atom parameters constrained
*R*[*F*^2^ > 2σ(*F*^2^)] = 0.037	*w* = 1/[σ^2^(*F*_o_^2^) + (0.0439*P*)^2^] where *P* = (*F*_o_^2^ + 2*F*_c_^2^)/3
*wR*(*F*^2^) = 0.088	(Δ/σ)_max_ = 0.001
*S* = 1.01	Δρ_max_ = 0.11 e Å^−^^3^
1540 reflections	Δρ_min_ = −0.13 e Å^−^^3^
148 parameters	Absolute structure: Flack *x* determined using 351 quotients [(*I*^+^)-(*I*^-^)]/[(*I*^+^)+(*I*^-^)] (Parsons *et al.*, 2013)
1 restraint	Absolute structure parameter: −1.7 (10)

### Special details

**Table:**

Geometry. All esds (except the esd in the dihedral angle between two l.s. planes) are estimated using the full covariance matrix. The cell esds are taken into account individually in the estimation of esds in distances, angles and torsion angles; correlations between esds in cell parameters are only used when they are defined by crystal symmetry. An approximate (isotropic) treatment of cell esds is used for estimating esds involving l.s. planes.

### Fractional atomic coordinates and isotropic or equivalent isotropic displacement parameters (Å^2^)

**Table:**

	*x*	*y*	*z*	*U*_iso_*/*U*_eq_	
O1	0.76287 (9)	0.3026 (5)	0.3893 (2)	0.0552 (6)	
N1	0.54406 (13)	−0.2364 (8)	0.5790 (3)	0.0652 (9)	
C1	0.58360 (13)	−0.1186 (7)	0.5054 (3)	0.0481 (9)	
O2	0.80114 (8)	0.9769 (6)	0.7178 (3)	0.0571 (6)	
H2	0.785835	1.067394	0.778171	0.086*	
C2	0.58576 (16)	−0.1796 (10)	0.3721 (4)	0.0696 (12)	
H2A	0.613875	−0.093141	0.322655	0.084*	
N2	0.54979 (15)	−0.3547 (9)	0.3114 (3)	0.0802 (11)	
N3	0.66429 (11)	0.1682 (6)	0.4996 (3)	0.0446 (6)	
C3	0.51042 (17)	−0.4693 (10)	0.3865 (5)	0.0718 (13)	
H3	0.483903	−0.592846	0.348009	0.086*	
N4	0.70225 (10)	0.3562 (6)	0.5568 (3)	0.0457 (7)	
H4	0.696761	0.411873	0.635489	0.055*	
C4	0.50756 (17)	−0.4124 (11)	0.5171 (4)	0.0766 (13)	
H4A	0.479179	−0.498671	0.565696	0.092*	
N5	0.76423 (10)	0.7824 (5)	0.6578 (3)	0.0447 (7)	
C5	0.62427 (13)	0.0788 (7)	0.5713 (3)	0.0467 (8)	
C6	0.61725 (16)	0.1548 (10)	0.7154 (4)	0.0674 (10)	
H6A	0.648915	0.087367	0.763582	0.101*	
H6B	0.585142	0.054609	0.748914	0.101*	
H6C	0.613240	0.370690	0.725479	0.101*	
C7	0.74946 (14)	0.4146 (6)	0.4956 (3)	0.0406 (7)	
C8	0.78699 (12)	0.6303 (7)	0.5654 (3)	0.0421 (7)	
C9	0.84549 (13)	0.6475 (9)	0.5264 (4)	0.0657 (11)	
H9A	0.853496	0.847820	0.494094	0.099*	
H9B	0.852677	0.501055	0.458348	0.099*	
H9C	0.868223	0.604687	0.601444	0.099*	

### Atomic displacement parameters (Å^2^)

**Table:**

	*U*^11^	*U*^22^	*U*^33^	*U*^12^	*U*^13^	*U*^23^
O1	0.0616 (13)	0.0633 (14)	0.0408 (14)	−0.0086 (12)	0.0065 (12)	−0.0188 (13)
N1	0.0593 (18)	0.086 (2)	0.0508 (18)	−0.0195 (17)	−0.0002 (17)	0.0000 (18)
C1	0.0450 (18)	0.0568 (19)	0.043 (2)	−0.0026 (16)	−0.0017 (17)	−0.0026 (18)
O2	0.0580 (13)	0.0618 (14)	0.0516 (14)	−0.0040 (12)	−0.0033 (13)	−0.0277 (12)
C2	0.065 (2)	0.094 (3)	0.050 (3)	−0.031 (2)	0.004 (2)	−0.011 (2)
N2	0.077 (2)	0.107 (3)	0.056 (2)	−0.033 (2)	−0.003 (2)	−0.016 (2)
N3	0.0455 (14)	0.0463 (14)	0.0422 (14)	−0.0036 (13)	−0.0015 (14)	−0.0097 (13)
C3	0.061 (2)	0.084 (3)	0.071 (3)	−0.022 (2)	−0.017 (2)	−0.002 (3)
N4	0.0499 (15)	0.0495 (15)	0.0377 (14)	−0.0044 (13)	0.0022 (15)	−0.0159 (13)
C4	0.063 (2)	0.103 (3)	0.064 (3)	−0.032 (2)	−0.006 (2)	0.011 (3)
N5	0.0538 (17)	0.0427 (13)	0.0375 (16)	0.0001 (13)	−0.0038 (14)	−0.0112 (13)
C5	0.0482 (18)	0.0527 (18)	0.0392 (18)	0.0012 (16)	−0.0016 (18)	−0.0059 (16)
C6	0.069 (2)	0.091 (3)	0.042 (2)	−0.012 (2)	0.005 (2)	−0.011 (2)
C7	0.0492 (17)	0.0382 (15)	0.0342 (19)	0.0017 (14)	0.0011 (16)	−0.0074 (16)
C8	0.0481 (16)	0.0432 (15)	0.0350 (17)	−0.0003 (14)	0.0021 (16)	−0.0053 (16)
C9	0.0555 (19)	0.080 (2)	0.062 (3)	−0.0123 (19)	0.014 (2)	−0.030 (2)

### Geometric parameters (Å, º)

**Table:**

O1—C7	1.229 (4)	N4—H4	0.8452
N1—C1	1.325 (4)	N4—C7	1.332 (4)
N1—C4	1.336 (5)	C4—H4A	0.9300
C1—C2	1.379 (5)	N5—C8	1.278 (4)
C1—C5	1.477 (4)	C5—C6	1.509 (5)
O2—H2	0.8200	C6—H6A	0.9600
O2—N5	1.382 (3)	C6—H6B	0.9600
C2—H2A	0.9300	C6—H6C	0.9600
C2—N2	1.319 (5)	C7—C8	1.496 (4)
N2—C3	1.325 (5)	C8—C9	1.482 (5)
N3—N4	1.370 (3)	C9—H9A	0.9600
N3—C5	1.279 (4)	C9—H9B	0.9600
C3—H3	0.9300	C9—H9C	0.9600
C3—C4	1.350 (6)		
			
C1—N1—C4	116.5 (3)	N3—C5—C1	115.8 (3)
N1—C1—C2	120.3 (3)	N3—C5—C6	124.7 (3)
N1—C1—C5	117.6 (3)	C5—C6—H6A	109.5
C2—C1—C5	122.2 (3)	C5—C6—H6B	109.5
N5—O2—H2	109.5	C5—C6—H6C	109.5
C1—C2—H2A	118.5	H6A—C6—H6B	109.5
N2—C2—C1	123.1 (4)	H6A—C6—H6C	109.5
N2—C2—H2A	118.5	H6B—C6—H6C	109.5
C2—N2—C3	115.8 (4)	O1—C7—N4	124.1 (3)
C5—N3—N4	117.4 (3)	O1—C7—C8	120.5 (3)
N2—C3—H3	119.0	N4—C7—C8	115.4 (3)
N2—C3—C4	122.1 (4)	N5—C8—C7	114.4 (3)
C4—C3—H3	119.0	N5—C8—C9	125.9 (3)
N3—N4—H4	117.9	C9—C8—C7	119.6 (3)
C7—N4—N3	120.1 (3)	C8—C9—H9A	109.5
C7—N4—H4	121.4	C8—C9—H9B	109.5
N1—C4—C3	122.3 (4)	C8—C9—H9C	109.5
N1—C4—H4A	118.9	H9A—C9—H9B	109.5
C3—C4—H4A	118.9	H9A—C9—H9C	109.5
C8—N5—O2	111.4 (2)	H9B—C9—H9C	109.5
C1—C5—C6	119.5 (3)		
			
O1—C7—C8—N5	165.1 (3)	N2—C3—C4—N1	0.2 (8)
O1—C7—C8—C9	−16.1 (5)	N3—N4—C7—O1	−2.1 (5)
N1—C1—C2—N2	−0.3 (7)	N3—N4—C7—C8	178.2 (2)
N1—C1—C5—N3	174.5 (3)	N4—N3—C5—C1	178.8 (2)
N1—C1—C5—C6	−4.0 (5)	N4—N3—C5—C6	−2.7 (5)
C1—N1—C4—C3	0.1 (6)	N4—C7—C8—N5	−15.2 (4)
C1—C2—N2—C3	0.6 (6)	N4—C7—C8—C9	163.6 (3)
O2—N5—C8—C7	−179.9 (3)	C4—N1—C1—C2	−0.1 (6)
O2—N5—C8—C9	1.4 (5)	C4—N1—C1—C5	179.5 (3)
C2—C1—C5—N3	−5.8 (5)	C5—C1—C2—N2	−179.9 (3)
C2—C1—C5—C6	175.6 (4)	C5—N3—N4—C7	168.4 (3)
C2—N2—C3—C4	−0.5 (7)		

### Hydrogen-bond geometry (Å, º)

**Table:**

*D*—H···*A*	*D*—H	H···*A*	*D*···*A*	*D*—H···*A*
O2—H2···O1^i^	0.82	1.94	2.741 (3)	167
C2—H2*A*···O2^ii^	0.93	2.35	3.243 (5)	161
C4—H4*A*···N2^iii^	0.93	2.67	3.451 (6)	142

Symmetry codes: (i) −*x*+3/2, *y*+1, *z*+1/2; (ii) −*x*+3/2, *y*−1, *z*−1/2; (iii) −*x*+1, −*y*−1, *z*+1/2.
